# Reversed-phase separation methods for glycan analysis

**DOI:** 10.1007/s00216-016-0073-0

**Published:** 2016-11-25

**Authors:** Gerda C. M. Vreeker, Manfred Wuhrer

**Affiliations:** 1 Center for Proteomics and Metabolomics, Leiden University Medical Center, PO Box 9600, 2300 RC Leiden, The Netherlands; 2 Division of Bioanalytical Chemistry, VU University Amsterdam, Faculty of Sciences, De Boelelaan 1083, 1081 HV Amsterdam, The Netherlands

**Keywords:** Glycan, Reversed phase, Liquid chromatography, Separation

## Abstract

Reversed-phase chromatography is a method that is often used for glycan separation. For this, glycans are often derivatized with a hydrophobic tag to achieve retention on hydrophobic stationary phases. The separation and elution order of glycans in reversed-phase chromatography is highly dependent on the hydrophobicity of the tag and the contribution of the glycan itself to the retention. The contribution of the different monosaccharides to the retention strongly depends on the position and linkage, and isomer separation may be achieved. The influence of sialic acids and fucoses on the retention of glycans is still incompletely understood and deserves further study. Analysis of complex samples may come with incomplete separation of glycan species, thereby complicating reversed-phase chromatography with fluorescence or UV detection, whereas coupling with mass spectrometry detection allows the resolution of complex mixtures. Depending on the column properties, eluents, and run time, separation of isomeric and isobaric structures can be accomplished with reversed-phase chromatography. Alternatively, porous graphitized carbon chromatography and hydrophilic interaction liquid chromatography are also able to separate isomeric and isobaric structures, generally without the necessity of glycan labeling. Hydrophilic interaction liquid chromatography, porous graphitized carbon chromatography, and reversed-phase chromatography all serve different research purposes and thus can be used for different research questions. A great advantage of reversed-phase chromatography is its broad distribution as it is used in virtually every bioanalytical research laboratory, making it an attracting platform for glycan analysis.

**Graphical Abstract:**
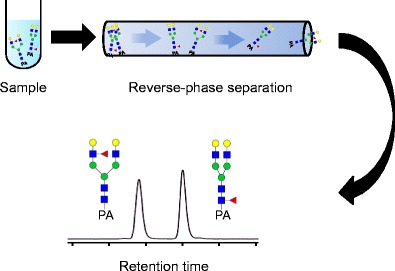
Glycan isomer separation by reversed phase liquid chromatography

Glycan isomer separation by reversed phase liquid chromatography

## Introduction

Glycosylation is a frequently observed posttranslational modification in proteins. Many membrane and secretory proteins are glycosylated while passing through the endoplasmic reticulum and Golgi system [1]. Glycans are composed of monosaccharides that contain many chiral centers and are connected by glycosidic linkages. They may have very complex three-dimensional structures [2], and stereoisomerism can have a substantial influence on the function of these molecules [3]. Various glycans are found in human cells; for example, *N*-glycans or *O*-glycans that are linked to proteins, next to lipid-linked glycans and free molecules [4]. Structural and conformational differences in proteins can be caused by glycans, which may result in modulated protein activity and protein interactions [3, 5, 6]. These molecules participate in many different biological processes, such as cell signaling and recognition, immune defense, and parasitic infections [3].

The analysis of protein glycosylation can be performed on different levels—intact glycoproteins [7, 8], glycopeptides [9–12], and released glycans [13–15]—each resulting in slightly different information on the glycoprotein. A disadvantage of the analysis of intact glycoproteins is that good separation of the different glycoforms of a glycoprotein is hard to achieve, especially for proteins with many glycosylation sites and in complex samples [3]. The analysis of glycopeptides has the advantage that the glycosylation can be assigned to specific locations on the protein. This site-specific information can be used to assign specific glycan structures to distinct glycosylation sites. Furthermore, it can contribute to the understanding of the molecular structure of the protein [9, 16].

In this review the focus is on released glycans. Glycans can be released from proteins and peptides in an enzymatic and chemical way [17, 18]. For *N*-glycans, various different enzymatic release methods are available, but for *O*-glycans, generally chemical release methods need to be used. The use of a chemical release method for glycans has several limitations: For example, the reducing-end aldehyde of the glycan can be reduced to an alditol by reductive β-elimination [19], thereby prohibiting subsequent labeling of the reducing end. After release, a derivatization step is often performed to improve the properties of the glycans for analysis. In addition, when one is working with complex biological samples, enrichment of the glycans needs to be performed. Hydrophilic interaction liquid chromatography (HILIC), graphitized carbon chromatography, and reversed-phase solid-phase extraction are the most used methods for enrichment of glycans [20–26]. Besides these methods, methods based on graphene have been developed [27, 28].

Information on the released glycans can be gathered with several different techniques. Separation techniques such as capillary electrophoresis (CE) and liquid chromatography (LC) are often used in combination with mass spectrometry (MS), fluorescence, or UV detection. In addition, matrix-assisted laser desorption/ionization MS is used for glycan analysis without separation or in combination with LC fractionation. Also, gel electrophoresis is a commonly used technique for glycan analysis. Most of these techniques can be used for analysis of glycans in both their native form and their derivatized form.

For released glycan analysis, various LC stationary phases are used, including high-pH anion-exchange [29, 30], HILIC [31–33], porous graphitized carbon (PGC) [34–36], and reversed-phase stationary phases (see Table 1). In high-pH anion exchange, deprotonation of hydroxyl groups is achieved, which contributes to the separation of the glycans. Both native and derivatized glycans can be separated with this technique [29]. PGC separation is based on hydrophobic and polar interactions [34, 37]. Native glycans are retained on the stationary phase and are eluted with water and acetonitrile [34]. Strong acidic or basic eluents can be used, because the columns are more hydrophobic and chemically stabler than reversed-phase columns [37].

**Table 1:** Reversed-phase (*RP*) liquid chromatography (*LC*) methods for glycan analysis

Column and flow	Solvents	pH	Samples	Derivatization	Separation	Detection	Ionization mode	Remarks	References	Year
RP nano column: C_18_ StableBond Zorbax 5 μm; 75 μm × 150 mm (0.250 μL/min)	Gradient of 97 % water–3 % acetonitrile–0.1 % formic acid with 0.5 mM sodium acetate and 97 % acetonitrile–3 % water–0.1 % formic acid with 0.5 mM sodium acetate	Acidic	*N-*Glycans from bovine fetuin and human blood serum samples	Reduction and permethylation	Separation was performed to minimize negative effects from competitive ionization	ESI-MS	+		[48]	2010
RP precolumn: Acclaim PepMap100 C_18_ nano trap column. Analytical RP column: Acclaim PepMap100 C_18_; 75 μm × 150 mm (0.300 μL/min)	Gradient of 2 % acetonitrile–0.1 % formic acid in water and 0.1 % formic acid in acetonitrile	Acidic	*N-*Glycans from model glycoproteins and from human blood serum	Reduction and permethylation	RP separation was used as the sample purification method	ESI-MS	+	Analysis of small amounts (low picomole to femtomolerange) is challenging	[49]	2011
RP nano column: PepMap; 75 μm × 150 mm (0.350 μL/min)	Gradient of 2 % acetonitrile in water with 0.1 % formic acid and acetonitrile with 0.1 % formic acid	Acidic	Released permethylated *N*-glycans from model glycoproteins (RNase B and porcine thyroglobulin) and human blood serum	Reduction and permethylation	Different glycan compositions were baseline-separated, but this was not the case for all samples	ESI-MS MALDI-TOF-MS	+	No detection of low-abundance structures	[43]	2012
RP precolumn: NanoEase Atlantis C_18_ 5 μm; 100 Å, 180 μm × 23.5 mm (10 μL/min). Analytical RP column: PepMap 100 3 μm; 75 μm × 150 mm (0.300 μL/min)	Gradient of 10 % acetonitrile in 0.1 % formic acid and sodium hydroxide and 90 % acetonitrile and 10 % 2-propanol in 0.1 % formic acid	Acidic	Purified glycan standards (sialyl Lewis X and sialyl Lewis A) and *N*-glycans originating from α-1-acid glycoprotein and IgG	Reduction and permethylation	Isomers of glycans were separated	ESI-MS	+	Sodium hydroxide was added to the eluent to induce sodium adduct formation. Glycans were analyzed in the low femtomole range	[44]	2013
Acclaim C_18_ nano column and HSS T3 C_18_ nano UPLC column (350 nL/min)	Gradient of 0.1 % formic acid in 2 % acetonitrile and 0.1 % formic acid in 100 % acetonitrile	Acidic	Released *N-*glycans from RNase B, fetuin, and human blood serum	Reduction and permethylation	Isomer separation was achieved at high temperatures	ESI-MS	+	Separation was performed at different temperatures: ambient to 75 °C	[50]	2016
Nano-LC RP trap column: PepMap 3 μm; 75 μm × 20 mm. RP nano column: Acclaim PepMap C_18_ 75 μm × 150 mm (300 nL/min)	Gradient of 0.1 % formic acid in 2 % acetonitrile and 0.1 % formic acid in 100 % acetonitrile	Acidic	Released *N-*glycans from RNase B, fetuin, and human blood serum	Reduction and permethylation	Glycans were not fully separated but were spread over a retention time range of 20–50 min. From MS-detection the different co-eluting glycans could be identified	ESI-MS	+		[52]	2016
Acclaim PepMap C_18_ 75 μm × 150 mm (350 nL/min)	Gradient of 0.1 % formic acid in 2 % acetonitrile and 0.1 % formic acid in 100 % acetonitrile	Acidic	Human, bovine and goat milk free oligosaccharides and *N*-glycans	Reduction and permethylation	Glycans were not fully separated but spread over a retention time range of 15 to 55 minutes. From MS detection the different coeluted glycans could be identified. Isomers of glycans were also separated	ESI-MS	+		[51]	2016
Alltech Adsorbosphere RP C_18_ column	Isocratic methanol–water (80:20) containing 1 % acetic acid	Acidic	Oligosaccharides	Permethylation	RP chromatography was only used to separate glycans from salt contaminants	ESI-MS	+		[121]	1997
Hypersil C_18_; 100 mm × 2.1 mm (0.2–0.4 mL/min)	Gradient and isocratic measurements with water and methanol and/or acetonitrile buffered with 1 mM sodium acetate	Acidic	Permethylated oligosaccharide mixtures	2-Aminobenzamide and permethylation	α and β anomers were differentiated in some cases, but in other measurements the separation of diantennary, triantennary and tetraantennary glycans was poor	ESI-MS	+		[122]	2001
Hypersil ODS C_18_ 3 μm; 150 mm × 4.6 mm (0.5–1.5 mL/min)	Gradient of 50 mM formic acid in water adjusted to pH 5 with triethylamine and 50:50 first solvent–acetonitrile	5.0	*O*-Glycans from bovine serum fetuin, human serum IgA1, human secretory IgA, human neutrophil gelatinase B, and human glycophorin A	2-Aminobenzamide	Low peak capacity, glycan species were not separated individually	FL (excitation 330 nm, emission 420 nm)		An ion-pairing reagent (triethylamine) was added to separate glycans containing sialic acids. Glycans were analyzed in the low femtomole range	[60]	2002
Hypersil ODS C_18_ 3 μm; 4.6 mm × 150 mm (0.5–1.5 mL/min)	Gradient of 50 mM formic acid in water adjusted to pH 5 with triethylamine and 50:50 first solvent–acetonitrile	5.0	*N*-Glycans and *O*-glycans from apolipoprotein (a)	2-Aminobenzamide	Glycans were separated but the run time was 180 min	FL (excitation 330 nm, emission 420 nm)			[84]	2001
Acquity UPLC BEH C_18_ 1.7 μm; 100 mm × 2.1 mm (0.350 mL/min)	Gradient of water and 25:75 methanol–water both containing 20 mM diethylamine (ion-pairing agent) and 50 mM formic acid	Acidic	Released *N*-glycans from monoclonal antibodies, fetuin, and RNase B	2-Aminobenzamide	Selectivity for glycans is low and low peak capacity	FL (excitation 250 nm, emission 428 nm)		An ion-pairing reagent (diethylamine) was added to separate glycans containing sialic acids	[61]	2011
Nano-LC RP trap column: PepMap 100 3 μm; 300 μm × 5 mm. RP nano column: PepMap C_18_ 100 3 μm; 75 μm × 150 mm (gradient pump 150 nL/min and microflow pump 10 μL/min)	Gradient pump: gradient of 0.4 % acetonitrile in water with 0.1 % formic acid and water–acetonitrile (5:95 v/v) containing 0.1 % formic acid. Microflow pump: 0.4 % acetonitrile in water with 0.1 % formic acid	4.4	Glycan pools	2-Aminobenzamide	ND	UV absorbance (254 nm) FL (excitation 360 nm, emission 425 nm) ESI-MS	+ and −	Protocol for RP separation only or as a second dimension after HILIC separation. Glycans were analyzed in the femtomole range	[38]	2009
Nano-LC RP guard column: PepMap; 300 μm × 10 mm. RP nano column: PepMap C_18_ 3 μm; 75 μm × 150 mm (150 nL/min)	Gradient of water–acetonitrile (95:5 v/v) containing 0.1 % formic acid and water–acetonitrile (5:95 v/v) containing 0.1 % formic acid	Acidic	Released glycans from glycoproteins from *Schistosoma mansoni* worms	2-Aminobenzamide	With RP separation in the second dimension after HILIC separation, the different glycans coeluted in HILIC were not fully separated	ESI-MS	+	Method for RP separation only and for separation in the second dimension after HILIC separation	[45]	2006
PepMap C_18_ 3 μm; 75 μm × 150 mm (150 nL/min)	Gradient of 0.8 mM sodium hydroxide in water–acetonitrile (95:5 v/v) containing 0.1 % formic acid and water–acetonitrile (5:95 v/v) containing 0.1 % formic acid	Acidic	Egg-derived oligosaccharides from urine from individuals infected with *S. mansoni*	2-Aminobenzamide	RP separation was used to obtain fragmentation spectra of major oligosaccharides	ESI-MS	+	Sodium hydroxide was added to the eluent to induce sodium adduct formation	[46]	2007
Nano-LC RP guard column: PepMap; 300 μm × 10 mm. RP nano column: PepMap C_18_ 3 μm; 75 μm × 150 mm (150 nL/min)	Gradient of 0.4 % acetonitrile in water with 0.1 % formic acid and water–acetonitrile (5:95 v/v) containing 0.1 % formic acid	Acidic	Released glycans from glycoproteins from *S. mansoni* worms and released *N*-glycans in tobacco plants	2-Aminobenzamide	Low peak capacity, elution of glycans was spread over time, but there was no clear separation. With RP separation in the second dimension after HILIC separation, the different glycans coeluted in HILIC were separated	ESI-MS MALDI-TOF	+	Method for RP separation only and for separation in the second dimension after HILIC separation	[47]	2006
Thermo Scientific C_18_ 3 μm; 250 mm × 4 mm (0.2 mL/min)	Gradient of water and 10:90 acetonitrile–water with both containing 0.1 % acetic acid	Acidic	Released *N*-glycans from recombinant IgG antibodies	2-Aminobenzamide	Glycans were separated but the run time was ≥140 min	FL (excitation 330 nm, emission 420 nm) ESI-MS	+	Glycans were analyzed in the femtomole range	[80]	2007
Thermo Scientific C_18_ 3 μm; 250 mm × 4 mm (0.2 mL/min)	Gradient of water and 10:90 acetonitrile–water with both containing 0.1 % acetic acid	Acidic	Released *N*-glycans from RNase B from bovine pancreas, ovalbumin (grade VII) from chicken egg, and fetuin from fetal calf serum	2-Aminobenzamide	Glycans were separated but the run time was ≥160 min	FL (excitation 330 nm, emission 420 nm) ESI-MS	+ and −	Glycans were analyzed in the femtomole range	[81]	2009
Waters T3 C_18_ 1.7 μm; 150 mm × 2.1 mm	Gradient of water and acetonitrile both containing 0.1 % formic acid	Acidic	*N*-Glycans from human, bovine, equine, and canine IgGs	2-Aminobenzamide	Different types of glycans were separated, but assignment of individual glycans was difficult. Sialylated glycans could not be separated	FL (excitation 330 nm, emission 420 nm)			[82]	2014
Zorbax rapid resolution SB-C18 1.8 μm; 50 mm × 2.1 mm (0.333 mL/min)	Gradient of water and 5 % acetonitrile in water both containing 0.1 % acetic acid	Acidic	Released *N*-glycans from recombinant IgG	2-Aminobenzamide	Separation of isomers was observed in a runtime of 50 min	FL ESI-MS	+	A rapid resolution column was used. The limit of detection was less than 10 fmol	[83]	2009
Xterra column C_18_ 3.5 μm; 2.1 mm × 150 mm (0.15 mL/min)	Gradient of 2 % acetonitrile in 0.1 % trifluoroacetic acid and 20 % acetonitrile in 0.1 % trifluoroacetic acid	Acidic	Purified oligosaccharides	2-Aminobenzamide	Isomers of glycans were separated but the run time was ≥180 min	UV absorbance (230 nm) ESI-MS	+		[125]	2005
Acquity UPLC BEH C_18_ 1.7 μm; 2.1 mm × 150 mm (0.3 mL/min)	Anthranilic acid: gradient of 1.0 % formic acid in water and 1.0 % formic acid in 50 % acetonitrile. 2-Aminobenzamide: gradient of 0.5 % formic acid in water and 0.5 % formic acid in 5 % acetonitrile	Acidic	*N*-Glycans from monoclonal antibodies	Anthranilic acid and 2-aminobenzamide	Isomers of glycans were separated but the run time was 80 minutes. Coelution of glycans was observed	FL (excitation 250 nm, emission 425 nm) ESI-MS	+		[126]	2013
Hypersil ODS column C_18_; 250 mm × 4 mm (1.2 mL/min)	Gradient of 50 mM ammonium formate and acetonitrile	4.4	Released *N*-glycans from bovine fibrin and IgG	2-Aminopyridine and other fluorescent labels for oligosaccharides	Desialylated IgG *N*-glycans can be separated, but this is strongly dependent on the label	MALDI-TOF-MS ESI-MS	+ and −	A less hydrophobic label increases the contribution of the glycan itself to the retention	[70]	2009
Shim-pack VP-ODS C_18_, 2 mm ID, and Shim-pack CLC-ODS C_18_, 6 mm ID	Gradient of water with 10 mM ammonium formate and water with 10 mM ammonium formate containing 0.5 % 1-butanol	4.0	Released *N*-glycans from human IgG from serum	2-Aminopyridine	Isomers of glycans were separated in a run time of 60 min	MALDI-TOF-MS			[88]	2009
Shim-pack HRC-ODS-silica C_18_; 150 mm × 6 mm (1.0 mL/min)	Gradient of 10 mM sodium phosphate buffer and 10 mM sodium phosphate buffer containing 0.5 % 1-butanol	3.8	Released *N*-glycans from human serum glycoproteins	2-Aminopyridine	Broad peaks and run time of ≥70 min, but separation of glycans	FL (excitation 320 nm, emission 400 nm) MALDI-TOF-MS	+		[89]	2007
Stainless steel column 4 mm × 250 mm packed with TSKgel (5 μm, C_18_). Cosmosil 5C18-P column (0.46 cm × 15 cm or 25 cm). μBondasphere 5 μm C_18_ 300 Å column (0.39 cm × 15 cm) (1.5 mL/min)	Gradient of 0.1 M ammonium acetate buffer and 0.1 M ammonium acetate buffer containing 0.5 % 1-butanol	4.0	*N*-Glycans	2-Aminopyridine	Different glycans were separated, but separation by gel filtration is needed before RP analysis (1986) [90, 91]. The method was used to purify glycans from glycoproteins (1990) [95]. Coelution of glycans was observed	FL (excitation 320 nm, emission 400 nm)		Prediction of retention times of glycans in RP chromatography	[90, 91, 95]	1986, 1990
Stainless steel column 4 mm × 250 mm packed with TSKgel (5 μm, C_18_) (1.6 mL/min)	0.1 M phosphate buffer	3.8	Glucose, lactose, laminaribiose, maltose, gentiobiose, cellobiose, and isomaltooligosaccharides	2-Aminopyridine	Isomers of glycans were separated, but not all glycans were baseline-separated	FL (excitation 320 nm, emission 400 nm)		Glycans were analyzed in the picomole range	[92]	1981
Cosmosil 5C18-P column (250 mm × 1.5 mm) (150 μL/min)	20 mM ammonium acetate buffer containing 0.075 % 1-butanol, with increasing concentration of 1-butanol during the run	4.0	*N*-Glycans	2-Aminopyridine	Coelution of glycans was observed	FL (excitation 320 nm, emission 400 nm)		Prediction of retention times of glycans in RP chromatography	[93]	1998
Shim-pack CLC-ODS-silica C_18_; 6 mm × 150 mm (1.0 mL/min)	ND	ND	*N*-Glycans from human IgG	2-Aminopyridine	Glycans were not all separated individually and the run time was ≥60 min	FL			[94]	2006
AquaSep C8 5 μm; 250 mm × 4.6 mm (1.0 mL/min)	Gradient of 0.05 % trifluoroacetic acid in water and acetonitrile	Acidic	Lactose and maltopentaose	2-Amino-5-bromopyridine	Low peak capacity	UV absorbance (200-320 nm) ESI-MS	+	The method was not optimized for RP separation. The limit of detection was approximately 14 pmol	[39]	2003
Various C_18_ phases with 3-μm particle size; 200 mm × 75 μm (0.3 mL/min)	Gradients of 5 mM ammonium acetate in water and acetonitrile	6.5	Dextrin 20, dextran from *Leuconostoc* ssp., glucose, and maltose	4-Aminobenzoic acid methyl ester, 4-aminobenzoic acid butyl ester, aminobenzoic acid ethyl ester, and 4-*n*-heptyloxyaniline	Low peak capacity, species were not separated individually	ESI-MS UV absorbance MALDI-TOF-MS	+		[59]	2002
Symmetry C_18_; 4.6 mm × 250 mm (1.0 L/min)	Gradient of 100 mM ammonium acetate (pH 6.69) and acetonitrile	Acidic	N-linked glycans released from α1-acid glycoprotein and IgG	2-Aminoacridone	Low peak capacity, species were not separated individually.	FL (excitation 442 nm, emission 520 nm) MALDI-TOF-MS	+		[100]	1997
Zorbax Eclipse XDB C_18_ 5 μm; 150 mm × 4.6 mm (1 mL/min)	Gradient of 0.1 M ammonium acetate in water and methanol	Acidic	Disaccharides from rat liver proteoglycans	2-Aminoacridone	Low peak capacity compared with SAX-HPLC, but labeled saccharides were separated	FL (excitation 425 nm, emission 520 nm)		Glycans were analyzed in the femtomole range	[102]	2008
Symmetry C_18_; 4.6 mm × 250 mm (1.0 L/min)	Gradient of 100 mM ammonium acetate and acetonitrile	6.6	Released glycans from bovine RNase B and α-acid glycoprotein and a dextran ladder	2-Aminoacridone and 3-(acetylamino)-6-aminoacridine	Low peak capacity, species were not separated individually	FL (excitation 442 nm, emission 525 nm) MALDI-TOF-MS	+ and −	Glycans were analyzed in the femtomole range	[103]	2000
Symmetry C_18_; 150 mm × 1.0 mm (0.050 mL/min)	Gradient of 10 mM triethylammonium acetate in water and 10 mM triethylammonium acetate in methanol	7.0	Released glycans from bovine fetuin, bovine RNase B, and chicken ovalbumin	8-Aminonaphthalene-1,3,6-trisulfonic acid	Low peak capacity, but separation of several isomers was achieved	UV absorbance (220, 262, and 354 nm) ESI-MS	−	Glycans were analyzed in the femtomole range	[104]	2003
Vydac 218-TP54 C_18_ 5 μm; 250 mm × 4.6 mm (1.0 mL/min)	Gradient of acetonitrile and water with a constant concentration of 0.04 % trifluoroacetic acid	Acidic	Released *N*-glycans from ovalbumin	1-Phenyl-3-methyl-5-pyrazolone	No efficient separation, sugars were eluted within the same range in 6 min	ESI-MS	+ and −	RP-HPLC was used as a desalting method because of inefficient separation. The detection limit was 2 nmol	[110]	2001
Vydac 218-TP54 C_18_ 5 μm; 250 mm × 4.6 mm (1.0 mL/min)	Gradient of 2:1 *tert*-butyl alcohol–acetonitrile and water with a constant concentration of 0.05 % trifluoroacetic acid	Acidic	*N*-Glycan standards	1-Phenyl-3-methyl-5-pyrazolone	Low peak capacity, but glycan standards were separated	ESI-MS	+		[108]	1999
Vydac 218-TP54 C_18_ 5 μm; 250 mm × 4.6 mm (1.0 mL/min)	Gradient of acetonitrile and water with a constant concentration of 0.04 % trifluoroacetic acid	Acidic	Tetraglucose and *N*-linked oligosaccharides	1-Phenyl-3-methyl-5-pyrazolone	ND	ESI-MS	+ and −	RP-HPLC was used as a desalting method. Glycans were analyzed in the picomole range	[134]	1999
Alltima C18-LL 5 μm; 150 mm × 2.1 mm. Guard column: Exsil C_18_ 5 μm; 2 mm (0.2 mL/min)	Gradient of acetonitrile–water in a ratio of 1:99 v/v and acetonitrile–water in a ratio of 80:20 v/v both containing 16 mM ammonium acetate, 24 mM acetic acid, and 0.5 mM triethylamine	4.5	Isolated oligosaccharides from MPS type IIIA urine	1-Phenyl-3-methyl-5-pyrazolone	Monosaccharides to octasaccharides were separated	UV absorbance (254 nm) ESI-MS	−		[107]	2006
Vydac 218-TP54 C_18_ 5 μm; 250 mm × 4.6 mm (0.3–1.0 mL/min)	First series: gradient of 0.05 M acetic acid in water and 0.05 M acetic acid in acetonitrile. Second series: gradient of water and 0.1 M acetic acid in 80 % acetonitrile in water	Acidic	Released *N*-glycans from hen ovalbumin (grades V and VII)	Phenylhydrazine	Low peak capacity, species were not separated individually	ESI-MS	+		[112]	2003
Vydac 218-TP54 C_18_ 5 μm; 250 mm × 4.6 mm (0.5 mL/min)	Gradient of 5 % acetonitrile in water and 90 % acetonitrile in water with 0.1 % trifluoroacetic acid	Acidic	Released *N*-glycans from mouse serum	Phenylhydrazine	Only used to separate types of glycans for better MALDI-MS analysis	UV absorbance (254 nm) MALDI-MS/MS	+		[113]	2008
Zorbax 300-SB C_8_ 5 μm; 150 mm × 4.6 mm (0.5 mL/min)	Gradient of 0.1 M acetic acid in 10:90 acetonitrile–water and 0.1 M acetic acid in 25:75 acetonitrile–water	7.0	Small saccharides: arabinose, galactose, glucose, GalNAc, GlcNAc, and lactose	Phenylhydrazine	Low peak capacity, but glycan standards were separated	UV absorbance (254 nm) ESI-MS	+	Glycans were analyzed in the picomole range	[40]	2003
μBondapak C_18_; 4 mm × 30 mm (2 mL/min)	Isocratic 22 % acetonitrile and 78 % water		Monosaccharides	Dansylhydrazine	Low peak capacity, species were not separated individually	UV absorbance (254 nm) FL (excitation 240 nm, emission 550 nm)		Glycans were analyzed in the nanomole range	[114]	1981

*BEH* bridged ethyl hybrid, *ESI* electrospray ionization, *FL* fluorescence, *GalNAc N*-acetylgalactosamine, *GlcNAc N*-acetylglucosamine, *HILIC* hydrophilic interaction liquid chromatography, *HPLC* high-performance liquid chromatography, *ID* internal diameter, *MALDI* matrix-assisted laser desorption/ionization, *MS* mass spectrometry, *MPS* mucopolysaccharidosis, *ND* no data, *ODS* octadecylsilyl, *SAX* strong anion exchange, *TOF* time of flight

The objective of this review is to compare reversed-phase separation methods for glycan analysis. An overview of the literature on this subject is presented, with the emphasis on separation of the glycans investigated. Various labeling compounds are compared for their advantages in separation and detection. In addition, the elution orders of the glycans are discussed.

## Column specifications and configurations

Reversed-phase chromatography is a widely used separation technique. An advantage of this technique is that it can be used in many laboratories, because only standard laboratory equipment is required [38]. In addition, various detection techniques can be used in combination with reversed-phase chromatography, depending on the labeling reagent used.

Reversed-phase separation is based on a noncovalent association between the nonpolar stationary phase and the nonpolar moieties of an analyte. The strength of this association depends on the polarity of the mobile phase [10]. The relative solubility of the analyte in the stationary phase and the mobile phase determines the degree of association of the analyte with the stationary phase and therefore the retention of the analyte. The retention is thus dependent on the competitive solubilization of the analyte between the stationary phase and the mobile phase.

An overview of the literature on reversed-phase separation of carbohydrates is presented in Table 1. As can be seen, in almost all methods a C_18_ reversed-phase column is used for separation. Only two of the methods use a C_8_ column to separate analytes [39, 40]. Although most methods are based on C_18_ separation, many different kinds of C_18_ columns are used. Reversed-phase chromatography is a commonly used analysis technique in chemistry and in other fields. Therefore an outstanding variety of C_18_ columns are commercially available. Columns with various different specifications are used. There are differences for example, in column length, internal diameter, and particle size, which may have a substantial influence on the separation efficiency. Differences between columns in terms of particle shape and bonded phase packing are illustrated by Snyder and Kirkland [41]. The hydrophobicity of the stationary phase also differs among columns [42]. In addition, the density and nature of the nonpolar groups immobilized on the silica surface will influence the selectivity [10].

Besides traditional and narrow-bore analytical reversed-phase columns, the use of analytical nanoscale reversed-phase columns is also described in several articles [38, 43–52]. Nano HPLC systems became commercially available in the 1990s. These nano HPLC columns typically have a dimension of 75 μm × 150 mm and a flow rate of around 300 nL/min. In addition, chip-based nano HPLC systems exist [53, 54]. Unfortunately, reduction of the internal diameter of the column will also limit the amount of sample that can be injected. To facilitate larger injection volumes, trapping columns are used. The analytes are trapped on a small column with relatively high flow rates and often large injection volumes followed by elution onto the longer analytical column for separation [55, 56]. By reduction of the internal diameter, the sensitivity of the measurements is increased with MS detection: sensitivities in the low femtomole range can be achieved in MS and MS/MS mode [53, 57, 58]. In addition, the use of nanoscale columns increases the separation efficiency and resolution, as was shown by Schmid et al. [59]. The coupling of nanoscale columns with MS detection is of importance for glycan analysis as the samples are often complex and full chromatographic separation is not obtained. Of note, separation of isobaric and isomeric glycan species is of particular value in combination with MS detection to achieve detailed characterization of complex glycan samples.

Full chromatographic separation, or at least the separation of isomers, can be achieved by use of two analytical columns. In work reported in a few articles, a reversed-phase column was used in the second dimension of two-dimensional LC [38, 45–47]. In the first dimension, separation was performed in HILIC mode at analytical scale, resulting in an incomplete separation of the different glycans in the samples. By use of nanoscale reversed phase in the second dimension, separation of complex samples and isomer mixtures was achieved. With this two-dimensional separation with nanoscale MS detection, detailed characterization of complex glycan mixtures can be achieved with high sensitivity.

## Solvents

As can be seen from Table 1, both gradient and isocratic runs are performed. In all cases the pH of the solvents is acidic or neutral. Most methods use a binary gradient, with solvent A consisting of mainly water and solvent B consisting of acetonitrile, methanol, or a mixture of one of these solvents with water. Solvent A may also contain up to 10 % acetonitrile. In addition, both solvents may contain low concentrations of a volatile acid (formic acid, acetic acid, or trifluoroacetic acid). Dependent on whether electrospray ionization (ESI) MS is used or not, volatile buffers (sodium acetate, triethylammonium acetate, ammonium acetate, or ammonium formate) or nonvolatile buffers (sodium phosphate) are used. In two cases, low amounts of sodium hydroxide were added to solvent A to induce sodium adduct formation in MS [44, 46]. In addition, in a few methods an ion-pairing reagent (diethylamine and triethylamine) is used to support retention of glycans with charged groups such as sialic acids [60, 61]. Dependent on which label was used, some adjustments in the solvents were made.

## Derivatization and detection

Although techniques that do not require derivatization exist [62], generally glycans are derivatized before reversed-phase separation and analysis. Carbohydrates absorb light only at low wavelengths, which results in low sensitivity in UV and fluorescence detection. In addition, amperometric and refractive index detection also have a problem with sensitivity when carbohydrates are analyzed. Coupling UV-absorbing and fluorescent molecules to these analytes greatly enhances the detection sensitivity in, for example, HPLC and CE [29]. Derivatization can also be used to increase detectability in MS by use of labels with a substituent that can be charged; for example, an amine or carboxylic acid group [29]. Also, derivatization with a hydrophobic label can increase sensitivity in ESI-MS, which was examined by Williams et al. [63]. However, a hydrophobic bias, which depends on the chemical and physical properties of the analytes, is present in ESI-MS [64]. In 1993, Fenn [65] described that ion desorption from droplets is dependent on the surface activity of the analyte. He compared this with the hydrophobicity of the analyte and showed in an example that the MS response increases with the number of carbon atoms per alkyl chain in tetraalkylammonium halides. This hydrophobic bias has consequences for quantitation by ESI-MS [66]. In addition, the signal intensity in ESI-MS depends on the amount of organic solvent present. For instance, the use of 80 % acetonitrile as the solvent showed a sixfold increase in intensity in the study of Bleicher and Bayer [67]. Moreover, the total signal intensity per analyte is also decreased by their being multiple charge states [68]. Quantitation by MS detection may for these reasons be compromised, unless an internal standard with properties similar to those of the analyte (e.g., an isotopically labeled version of the analyte) is used for absolute quantitation [69].

**Fig. 1:**
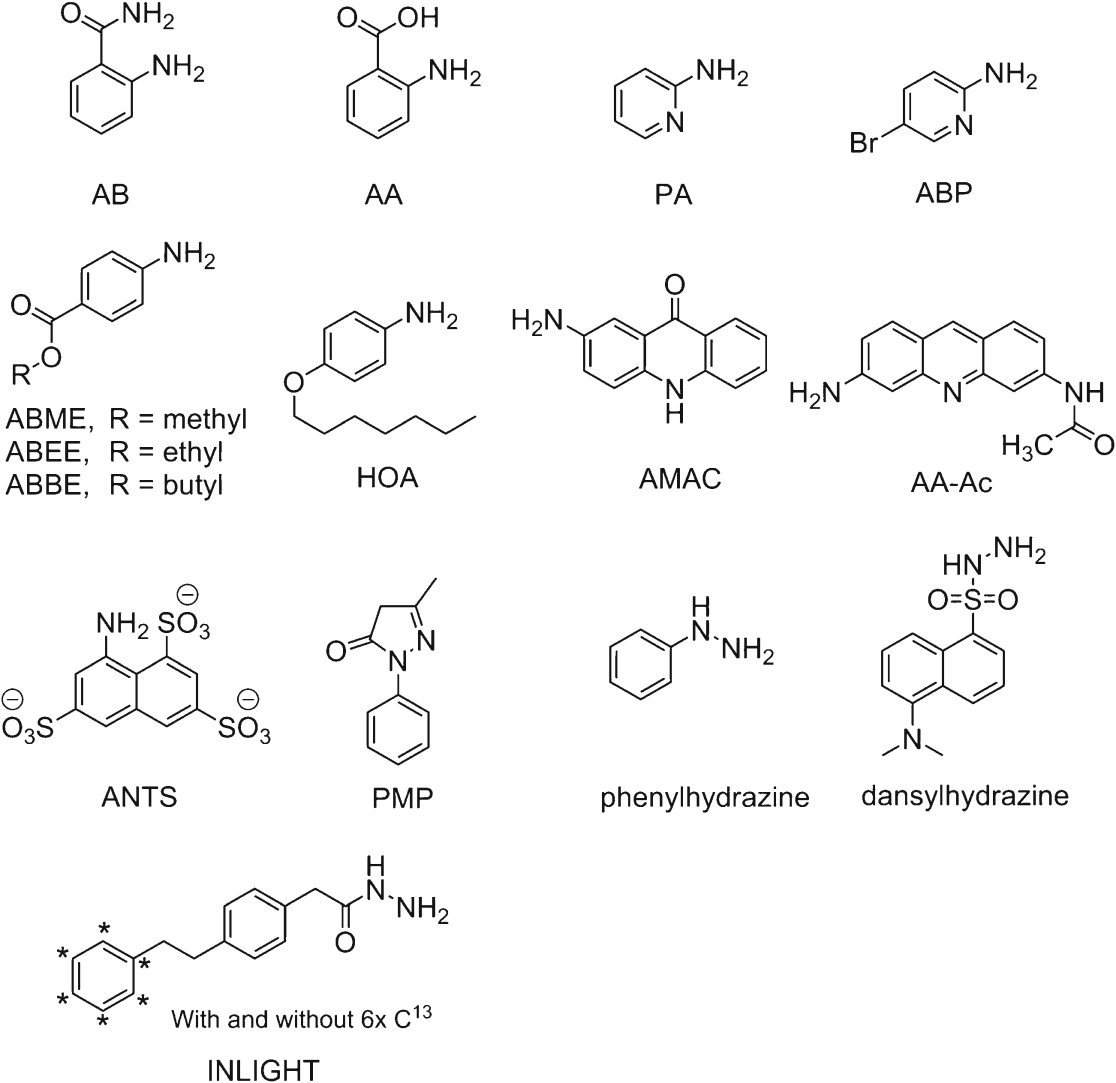
Structures of labels used in reversed-phase chromatography of oligosaccharides. *AA* anthranilic acid, *AA-Ac* 3-(acetylamino)-6-aminoacridine, *AB* 2-aminobenzamide, *ABBE* 4-aminobenzoic acid butyl ester, *ABEE* 4-aminobenzoic acid ethyl ester, *ABME* >4-aminobenzoic acid methyl ester, *ABP* 2-amino-5-bromopyridine, *AMAC* 2-aminoacridone, *ANTS* 2-aminonapthalene trisulfone, *HOA* 4-*n*-heptyloxyaniline, *INLIGHT* individuality normalization when labeling with isotopic glycan hydrazide tags, *PA* 2-aminopyridine, *PMP* 1-phenyl-3-methyl-5-pyrazolone

To be able to analyze carbohydrates with reversed-phase chromatography, it is important to make the analytes more hydrophobic so they can interact with the alkyl chains in the stationary phase. As shown in Table 1, various labels are used in reversed-phase chromatography of glycans: 2-aminobenzamide (AB), anthranilic acid (AA), 2-aminopyridine (PA), 2-amino-5-bromopyridine (ABP), 4-aminobenzoic acid methyl ester (ABME), 4-aminobenzoic acid ethyl ester (ABEE), 4-aminobenzoic acid butyl ester (ABBE), 4-*n*-heptyloxyaniline (HOA), 2-aminoacridone (AMAC), 3-(acetylamino)-6-aminoacridine (AA-Ac), 2-aminonapthalene trisulfone (ANTS), 1-phenyl-3-methyl-5-pyrazolone (PMP), phenylhydrazine, dansylhydrazine, and the individuality normalization when labeling with isotopic glycan hydrazide tags (INLIGHT). The structures of these labels are shown in Fig. 1. Besides labeling of the reducing end, reduction of the reducing end and permethylation of the glycan were also performed (Table 1). Another reducing-end label gaining popularity for HILIC analysis with fluorescence detection is procainamide [70, 71], yet its use in reverse-phase chromatography has still to be established. Likewise, labels that target glycosylamines generated by PNGase F release of *N*-glycans such as InstantAB^TM^ [72, 73] may be suitable for reversed-phase separation of glycans yet these analyses have still to be developed. Recently isotopic aminoxyTMT labels were introduced by Afiuni-Zadeh et al. [74], and these can be used for quantitation of peptides as well as glycans in MS. Zhou et al. [75] used these labels for glycan analysis on a PGC column, and stated that the label was not hydrophobic enough to obtain effective separation on a reversed-phase column.

Most labels described in Fig. 1 can be coupled to glycans by reductive amination, which is the most commonly used glycan derivatization technique. In this reaction, first a Schiff-base intermediate is formed. This Schiff base is subsequently reduced with sodium cyanoborohydride or 2-picoline borane to form a stable secondary amine [76, 77]. This derivatization reaction is often performed in methanol or dimethyl sulfoxide with acetic acid added to the organic solvent [78]. An important feature of this method is the stoichiometric coupling of one label per glycan, which together with the usually high labeling efficacies makes quantitation by fluorescence or UV detection possible [29]. The reaction mechanism for Schiff-base formation was described by Anumula [79], who showed that the labeling reaction is initiated by the attack of the lone pair of the amino group of the label on the carbon of the aldehyde of the reducing end of the glycan.

Various molecules containing an amino group can be coupled to glycans by reductive amination. The most commonly used label for glycan analysis is AB, which is used for the analysis of *N*-glycans [61, 80–84] as well as *O*-glycans [60, 84]. This label can be combined with various glycoanalytical methods, including several chromatographic phases (e.g., HILIC and reversed phase) and detection methods (e.g. UV detectors, fluorescence, and MS) [85, 86]. AA is a label that is similar to AB. This label contains a carboxylic acid moiety instead of the amide group. Labeling with AA provides at least two times more sensitivity in fluorescence detection as compared with labeling with AB [79]. In addition, AB and AA can both be used in positive and negative ion mode MS [32, 38, 81]. The labeling procedures for AA and AB labels were optimized by Bigge et al. [87] in the 1990s. They measured a labeling efficiency of more than 80 % for AB and approximately 80 % for AA. These labeling efficiencies were also investigated by Ruhaak et al. [77], who reported that, with sodium cyanoborohydride and 2-picoline borane as reducing agents, in most cases an almost complete labeling of glucose polymers was obtained.

Another label that is often used for labeling at the reducing end by reductive amination is PA [70, 88–95]. Hase et al. [92] were the first to use PA labeling for HPLC analysis of glycans. PA has a relatively low hydrophobicity compared with the other labels mentioned, which results in a rapid desorption from the stationary phase, providing sharp elution peaks [96]. In many of the methods using PA, a buffer was added to the solvents to maintain a low pH. As the p*K*_a_ of PA is 6.8, the molecule is protonated at low pH [97]. Testa and Wild [98] showed that this protonation results in increased fluorescence. This is beneficial for fluorescence detection, which is often used in combination with this label (Table 1). As a consequence of the low hydrophobicity of the label, low concentrations of organic solvents are used in the eluents, which makes direct coupling with ESI-MS less convenient [70]. ABP is PA with an additional bromine moiety. This bromine is advantageous when MS analysis is used, because of the natural equal abundances of ^79^Br and ^81^Br. In high-resolution MS, signals resulting from labeled species can easily be recognized by the typical isotopic pattern [39].

ABME, ABEE, ABBE, and HOA are less commonly used glycan derivatization reagents. These labels were compared by Schmid et al. [59], who showed that the retention times increased with a longer alkyl chain at the 4-position of the label. The polarities of the glycans tend to be very similar after derivatization with ABME, resulting in rapid elution and poor resolution for large glycans. Longer retention and better separation were observed for the other three labels [59, 99]. Sensitivity increases for positive and negative mode ESI-MS were tested by Pabst et al. [70], who showed that in positive mode, ABEE increased sensitivity by a factor of 2 compared with the native glycans. In negative mode a slight increase in sensitivity was observed with ABEE and ABBE.

AMAC is a labeling reagent with pronounced hydrophobic properties [100]. The molecule has fluorescent properties and a strong UV absorbance, which can be used for fluorescence analysis [101, 102]. Nevertheless, the fluorescence sensitivity of AMAC was somewhat less than that of AB and around four times than that of AA [32]. Charlwood et al. [103] reported AA-Ac based on AMAC as a new derivatization reagent. This label has higher fluorescence intensity than AMAC [103]. In positive ion mode ESI-MS, an intensity gain of a factor 2 was observed for AA-Ac compared with AA, whereas for AMAC the intensity was considerably lower [70].

ANTS is a charged molecule, which means that the label is hardly retained in reversed-phase chromatography [104]. Although it might not seem a natural choice to use this charged label in reversed-phase chromatography, Gennaro et al. [104] used it to separate glycans with a sensitivity in the low femtomole range. An ion-pairing reagent, triethylammonium acetate, was added to the mobile phase to create neutral complexes with the label, which then could be retained by the reversed-phase stationary phase. Like ANTS, 8-aminopyrene-1,3,6-trisulfonic acid, which is similar to ANTS, has been used for glycan separation on a reverse-phase stationary phase [105]. Glycans derivatized with ANTS were reported to have increased sensitivity in ESI-MS as compared with native glycans [104]. However, this increase was not observed by Pabst et al. [70].

ANTS was first used as a fluorophore in gel electrophoresis. In addition, it is used as a derivatizing reagent for CE and HILIC separations [29, 106]. Analysis of ANTS derivatives by reversed-phase chromatography can provide information on isomers and can be used as a technique complementary to CE and HILIC [104]. Reversed-phase separation of ANTS-derivatized glycans was obtained with an ion-pairing reagent. Without this ion-pairing reagent, ANTS-labeled analytes are found in the flow-through [104]. Various labels coupled to the reducing end by reductive amination were compared by Pabst et al. [70]. Unfortunately the derivatization protocols were not optimized, and thus no labeling efficiencies were shown.

PMP is a label that is added with a stoichiometry of two labels per glycan [107]. This addition of two labels might be a disadvantage, as it leads to a rather bulky reducing-end modification that may dominate separation. The labeling reaction with PMP is a Michael addition, which is performed under alkaline conditions [29]. In these alkaline conditions, loss of the sialic acids during derivatization is prevented [108]. PMP is a UV-absorbing molecule, but does not have fluorescent properties [29, 78]. In positive ion mode in ESI-MS, the sensitivity is approximately double that of native glycans, whereas in negative mode this gain was not observed [70]. PMP has been used for CE, HILIC and reversed-phase chromatography separations of glycans [109–111]. Saba et al. [110] observed in reversed-phase chromatography an elution range of only 6 min for the *N*-glycans of ovalbumin, whereas for HILIC the glycans were eluted over a range of 35 min. In addition, in-source fragments were observed in reversed phase LC–MS, whereas these were not observed with HILIC coupling. For these reasons, it was concluded that reversed-phase conditions were more useful for desalting PMP-derivatized samples than as a separation method [110, 112].

Derivatization of glycans can also be performed with hydrazine labels [40, 112–114]. The carbonyl group of the reducing-end aldehyde reacts with the hydrazine moiety to form a hydrazone bond [78]. This reaction is relatively clean, because no salts are used or produced during the reaction, which makes sample cleanup after the reaction often not necessary [40]. High labeling efficiencies for phenylhydrazines were observed for this reaction by Lattova and Perrault [40]. Dependent on the analysis technique, various hydrazine-containing molecules can be coupled to glycans; for example, dansylhydrazine, which has fluorescent properties and thus can be used in fluorescence detection [114]. Another hydrazine label for glycan analysis, the INLIGHT label, was introduced by Walker et al. [115] in 2011. There are two variants of this label, one of which contains six ^13^C atoms and the other one does not. The use of both labels makes quantification by MS possible. These labels can be used in combination with HILIC separation [116, 117], but reversed-phase separations are also performed [118, 119]. Unfortunately, no information was given on the separation efficiency of glycans with these labels.

In native glycans, the monosaccharide at the reducing-end terminus equilibrates between an open-ring conformation and a closed-ring conformation [2]. In the closed-ring conformation the C-1 atom is a chiral center with α and β anomers. In chromatography, small differences or double peaks could be observed for saccharides with a degree of polymerization of three monomers or more, because of these α and β anomers in the molecules. To overcome this without further labeling of the glycan, the reducing-end aldehyde in the open-ring conformation can be reduced with a reducing agent such as sodium borohydride, to form an alditol [29, 44, 120]. Other methods to eliminate double peaks (e.g., increasing the column temperature) exist but they will not be discussed in this review [10].

Glycans can also be derivatized by permethylation [43, 44, 48, 121, 122]. In contrast to the other derivatization methods discussed in this review, this derivatization does not occur at the reducing end. The hydrogens on the oxygen and nitrogen atoms in the glycans are replaced by methyl moieties, resulting in a largely hydrophobic molecule [122, 123]. This change in polarity and hydrophobicity is beneficial for glycan analysis with reversed-phase chromatography. In addition, permethylated compounds show an increase in detection sensitivity in ESI-MS, but detection in the femtomole range is challenging [48, 49, 122]. Notably, sialic acids are stabilized by permethylation via esterification, thereby preventing the ready loss of sialic acids observed in matrix-assisted laser desorption/ionization time-of-flight MS analysis of native glycans [124].

## The influence of reducing-end labels on glycan retention

**Fig. 2:**
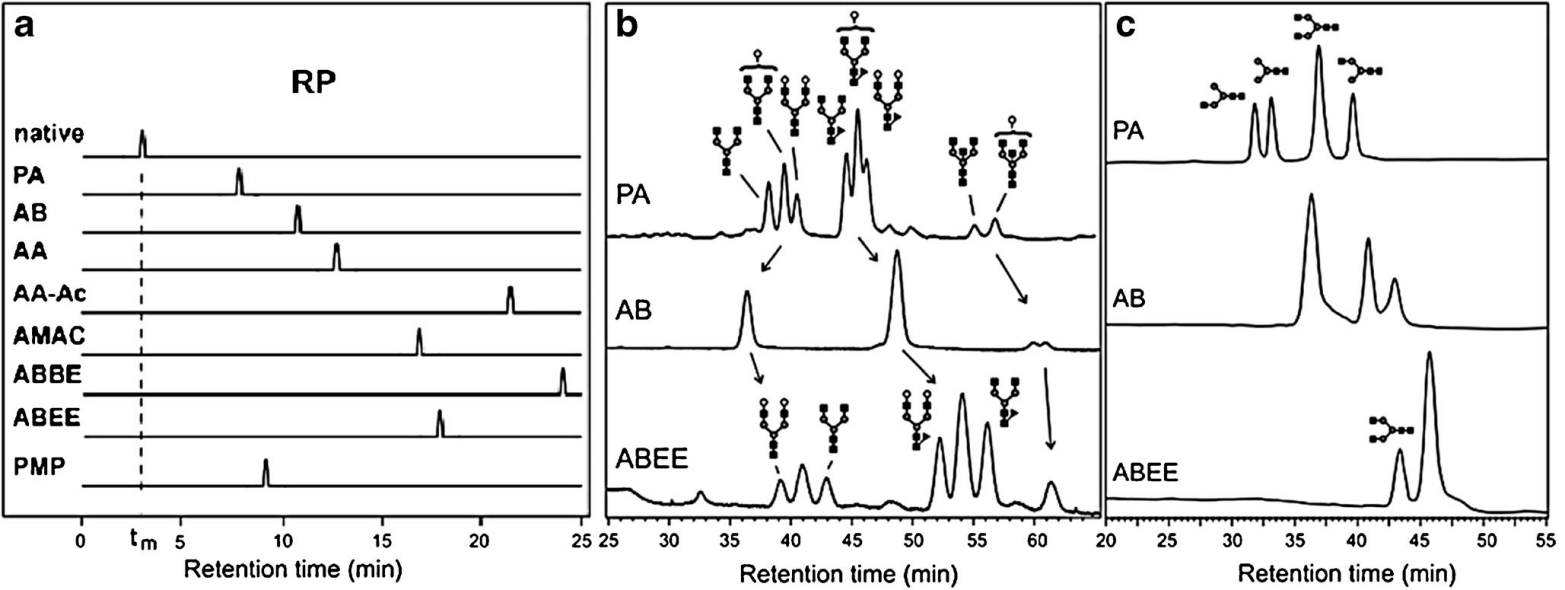
**a** Overview of the chromatograms of various reducing end derivatized *N*-glycans and a native glycan that is eluted at the void volume (*dashed line*). **b** Reversed-phase chromatograms of desialylated immunoglobulin G *N*-glycans derivatized with PA, AB, or ABEE. **c** Chromatograms of the separation of a mixture of four glycans: (GlcNAc)_2_(Man)_3_(GlcNAc)_2_, (GlcNAc)_2_(Man)_3_, and two isomers of (GlcNAc)_2_(Man)_3_(GlcNAc), where GlcNAc is *N*-acetylglucosamine and Man is mannose. *RP* reversed phase. (Reproduced and modified from Pabst et al. [70] with permission)

Glycans are hydrophilic molecules that tend to be hardly retained on many reversed-phase materials. Various labeling techniques may help to achieve glycan retention reversed-phase separation. An overview of the retention of native glycans as well as some labeled glycans is given in Fig. 2a. PA is the least hydrophobic of the commonly used labels and thus has a relatively low interaction with the stationary phase. The weak interactions of this label with the stationary phase results in the contribution of the glycan itself to the retention being relatively high with the PA tag compared with more hydrophobic tags [70]; therefore structural information can be derived from the glycans derivatized with this label. With the small, low-hydrophobicity PA label, specific glycan structural details tend to have relatively large influences on separation, which results in better separation of different species, as was shown by Pabst et al. [70]. They compared the separation of PA-, AB- and ABEE-labeled immunoglobulin G glycans, which is shown in Fig. 2b and c. In the case of PA-labeled glycans, small complex glycans were eluted first, whereas for ABEE-labeled glycans, the larger complex glycans were eluted first. This can be explained by the aforementioned contribution of glycans to the retention. In the case of PA, the retention is for a large part dependent on the interaction of the glycan with the stationary phase. Thus if the glycan has a higher degree of polymerization, it may engage in more interactions, resulting in longer retention. In the case of ABEE, the retention is mainly based on the hydrophobic label and the size of the hydrophilic glycan. One may speculate that the glycan interferes with the interaction of ABEE and the stationary phase, thereby leading to earlier elution of glycans with increasing degree of polymerization. For AB-labeled glycans, the glycan hardly modulates the retention contribution of the tag, resulting in a relatively poor reversed-phase separation of glycans [70]. Nevertheless, the separation of AB-labeled glycans can still be accomplished, with use of long gradients [80, 84, 125]. In this separation a negative correlation between the retention and the size of the high-mannose glycans was observed, whereas for complex glycans this correlation was positive [61, 126].

Schmid et al. [59] investigated on the separation of glycans labeled with ABME, ABEE, ABBE, and HOA. The first three labels mentioned differ by the length of the alkyl chain at the *para* position from the amine, and HOA contains an *n*-heptoxyl group at the *para* position. It was shown that this small difference in chain length had a substantial influence on the separation of the glycans and the run time per sample. The retention times are longer for ABEE and ABBE than for ABME because these labels are more hydrophobic. The longest retention times were measured for HOA. Better separations, including baseline-separated peaks, were obtained for ABEE, ABBE, and HOA.

Besides the different labels, Schmid et al. also tested different C_18_ stationary phases (i.e., differences in end-capping, pore size, etc.) for the separation of ABBE-labeled glycans, and showed differences in separation and retention times. One of these stationary phases was, for example, coated with a reactive polymeric silicone film that chemically binds to the silica gel and is alkylated afterward. For this encapsulated stationary phase, baseline separation of most of the analytes was observed, whereas for the partially trimethylsilyl end-capped stationary phase, overlap between all analytes was observed [59]. Furthermore, Gillmeister et al. [88] compared columns from various manufacturers, and showed major differences in retention and separation of glycans. The choice of the C_18_ column for separation can thus have a substantial influence on the separation efficiency.

## Separation of permethylated glycans

Permethylation of glycans has also regularly been used as a derivatization technique followed by reversed-phase LC–MS (Table 1). In this case the glycan itself is made less hydrophilic by the substitution of hydrogen atoms for methyl groups. This separation is thus based on the properties of the glycan and results in the smallest glycans being eluted first. Coelution of permethylated glycans is observed for the more complex samples, but because of MS detection these overlapping glycan species can still be identified [43, 51, 52]. Ritamo et al. [44] and Zhou et al. [50] observed separation of isomers, although in some cases the sample complexity was limited. The latter researchers performed the separation of permethylated glycans at higher temperature, which increased the chromatographic resolution of isomers and improved the peak shape and decreased the peak width. In addition, it was concluded that the influence of the three-dimensional structure of the glycans was reduced because of the higher-temperature separations, resulting in more predictable retention times, which could be beneficial for the identification of glycans.

## Glycan structural features influencing retention

AB labeling is often used in LC–MS methods. The separation of glycans labeled with AB was studied by Prater et al. [83] and Higel et al. [126], who showed that oligomannose glycans are eluted first. The main separation of the complex and hybrid glycans is caused by the core fucose: glycans containing a core fucose are eluted later than glycans without core fucosylation. Within these two groups, the acidic hybrid and complex glycans are eluted before the neutral hybrid and complex glycans, with the hybrid glycans being eluted before the complex ones. For AA labeling this elution order is almost the same [126]. Unfortunately, no information on isomers, bisecting variants, or triantennary variants was presented. Notably, when an ion-pairing reagent is used in combination with AB-labeled glycans the retention time of the sialylated glycans increases, which results in elution of sialylated glycans after neutral glycans [61]. Chen and Flynn [80] also performed reversed-phase separation of AB-labeled *N*-glycans, and found a slightly different elution order of the different types of glycans. They described that fucosylated sialylated glycans are eluted first from the column, followed by high-mannose glycans and neutral complex glycans [80, 81]. The difference between the elution order described by Higel et al. and this elution order might be due to differences in the columns, eluents, and gradients used, but this was not investigated. In addition Chen and Flynn [80] also described the elution of fucosylated triantennary and tetraantennary glycans. With their method these triantennary and tetraantennary glycans are eluted after the nonfucosylated glycans and before fucosylated diantennary species.

PA-labeled glycans show an increased relative retention of glycans containing a higher number of sialic acids [127, 128]. In contrast, for PMP-derivatized glycans the sialylated glycans are eluted earlier than (GlcNAc)_2_(Man)_3_, where GlcNAc is *N*-acetylglucosamine and Man is mannose, but in this case it cannot be said if this was due to the size of the glycans or the sialic acids present [108]. Tomiya and Takahashi [128] determined the elution positions of various neutral and sialylated PA-derivatized glycans. They calculated the contributions of the different monosaccharides at various positions in the *N*-glycan, taking linkages into account. These contributions were expressed in glucose units, which can be calculated by comparison of the retention times of the glycans with the retention times of the standard PA-isomaltooligosaccharides or a standard dextran ladder. The advantage of using glucose units instead of retention times is that glucose units are largely independent of the system and column used, which makes glucose units highly reproducible [60, 128].

The contribution of sialic acids to the retention on a C_18_ stationary phase strongly depends on the linkage (e.g. α2,3 linkage vs α2,6 linkage) and also on the type of sialic acid present (e.g., *N*-acetylneuraminic acid or *N*-glycolylneuraminic acid). In addition, the retention is also highly dependent on which antenna is sialylated [128]. For glycopeptides the elution order of neutral and sialylated species is the opposite: neutral glycan chains are eluted earlier than sialylated glycans with the same peptide moiety. Glycopeptides containing two sialic acid moieties on their glycans are eluted even later [129]. It appears that the different contributions of sialic acids to the retention of reducing-end labeled glycans as well as glycopeptides is still incompletely understood.

Besides the presence of sialic acids, also bisection of the glycan can influence the retention on the reversed-phase stationary phase. The addition of a bisecting GlcNAc to PA-labeled glycans results in a strong positive contribution to the retention [128]. The retention time increases even more if the bisecting GlcNAc contains a β1,4-linked galactose, which is a glycan structure that was discovered in immunoglobulin G by Takegawa et al. [130].

The separation of AMAC-labeled glycans was described by Okafo et al. [100]. The elution order of glycans was comparable to the elution order of the other relatively more hydrophobic labels, thus with the tetraantennary glycans being eluted before the triantennary glycans, which are eluted before the diantennary glycans. Okafo et al. [100] also found that antenna fucosylated glycans were eluted earlier than their nonfucosylated analogues, whereas the core-fucosylated glycans showed more retention than their nonfucosylated analogues.

This elution order of fucosylated glycans was observed not only for AMAC-labeled glycans but also for AB- and PA-labeled glycans [47, 128]. Tomiya and Takahashi [128] showed with their calculations on PA-labeled glycans that a core fucose on a *N*-glycan can have a major effect on the retention of the glycan on a reversed-phase stationary phase. This effect again depends on the linkage of this fucose (e.g., α1,6-linked fucose or α1,3-linked fucose). In vertebrate *N*-glycans, mainly α1,6-linked core fucoses are present, and these fucoses have a large positive contribution to the retention on a C_18_ stationary phase. Furthermore, the contribution of antenna fucosylation was also calculated. This contribution again depends on on which antenna the fucose is located and the linkage to this antenna. It was calculated that all antenna fucoses have no or a negative contribution to the retention in a C_18_ stationary phase [128]. This difference in retention of core and antenna fucoses might be explained by the polarity of a fucose. Fucoses are relatively apolar monosaccharides compared with the other monosaccharides present in *N*-glycans, because of their methyl group. In core-fucosylated glycans, the fucose is located relatively close to the label that interacts with the stationary phase, and both may interact concertedly with the stationary phase, leading to increased retention. In contrast, when the fucose is positioned on one of the antennae such a concerted interaction is not likely, which may explain the slightly lower retention of antenna-fucosylated glycans as compared with core-fucosylated glycans [47, 100, 128]. In PGC analysis of fucosylated glycans it was similarly found that antenna-fucosylated glycans are eluted before core-fucosylated glycans [131]. The different contribution of antenna and core fucoses to the retention is minor when a HILIC stationary phase (e.g., an amide column) is used because both types of fucosylation have a similar positive contribution to the retention [71, 128]. Notably, in glycopeptides also an effect in reversed-phase retention is observed when a core fucose is present. This effect again depends on the linkage of the fucose: when the fucose is α1,6-linked, it has a positive contribution to the retention and when the fucose is α1,3-linked, it has a negative contribution to the retention [132].

## Toward full resolution of glycan isomers

As can be seen from Table 1, the ability to separate glycans depends highly on the complexity of the sample. When the complexity of the sample is high, separation of all structures is often not obtained. However, for glycan standards containing fewer glycan species and small saccharides, separation of the different glycan species and isomers was observed.

A low peak capacity is observed in many of the methods mentioned. This clustering of peaks can be expected because in most cases the label has a major influence on the retention. In addition, the structural difference between the glycans is often small and has only a minor influence on the retention, resulting in low selectivity. For these reasons, high-resolution glycan separations are often not obtained. To spread the signals, longer run times can be used for separation. However, this will also increase the turnaround time of the method. In addition, resolution can be increased by use of ultra-high-performance LC, where separation is performed at pressures above 400 bar [83, 133]. Another option to obtain full separation of glycans is the addition of a second-dimension separation, as mentioned before [38, 45, 47]. A protocol for the separation of AB-labeled glycans with HILIC separation in the first dimension and reversed-phase separation in the second dimension has been described [38]. An advantage of this approach is that with these two HPLC dimensions, isobaric and isomeric structures can often be separated, which is beneficial for the characterization of individual glycans.

Notably, reversed-phase chromatography is sometimes used to desalt the sample or to create a rough separation of highly complex samples and not to obtain fully separated glycans [49, 110, 113, 121, 134]. This full separation of the various glycans is often not necessary when MS detection is used, because ions of a specific *m*/*z* can be selected for further analysis or to obtain an extracted ion chromatogram. However, the separation of isomeric and isobaric species is of particular relevance for glycan analysis. With use of ANTS, a (Glc)_3210_ maltooligosaccharide ladder was successfully separated by Gennaro et al. [104]. In addition, high-mannose *N*-glycan structures (GlcNAc)_2_(Man)_5–9_ from ribonuclease B and three isomers of Man_7_ and two isomers of Man_8_ were separated. However, the separation of the high-mannose structures was only visible with MS detection, because the different high-mannose species (Man_5–9_) are eluted with an overlap within 4 min. Unfortunately, in UV or fluorescence detection a distinction between overlapping peaks cannot be made. Full separation of glycan species and isomers is thus desired, which can be a challenge for complex samples containing many glycan species.

Other stationary phases might also be of use for the separation of glycan isomers. Similarly to reversed-phase chromatography, PGC chromatography is based on hydrophobic interactions with the analytes. However, in PGC chromatography polar and ionic interactions are also involved in the retention [34, 37]. It should be noted that the exact retention mechanisms of PGC chromatography are debatable [127]. The polar and ionic interactions with the analytes mean that native glycans can be separated by PGC chromatography [131]. This separation can be performed on the basis of the branching, sequence, and linkage of the glycans and therefore PGC chromatography is highly suitable for the separation of isomers [13, 135]. For example, Thaysen-Andersen et al. [127] reported on the linkage-specific retention of α2,3- and α2,6-linked sialylated glycans, showing later retention of α2,3-linked sialylated diantennary and triantennary glycans. Nevertheless, the robustness and reproducibility of PGC chromatography is limited [61]. PGC chromatography and HILIC have been found to be more powerful in separating glycan structural isomers than reversed-phase chromatography of the AB-labeled variants [127]. However, reversed-phase separation of *N*-glycans is generally more efficient than HILIC separation, as was discussed by Walker et al. [136]. The different stationary phases might be used for different purposes, as was mentioned by Melmer et al. [61]. HILIC was described as a useful method for the analysis of complex samples, because of its high peak capacity and PGC chromatography was described as a useful method for separation of isomers. Reversed-phase chromatography was described as a useful method for quality control purposes, because of its reproducibility.

## Conclusion

Reversed-phase chromatography is an technique often used for the separation of glycans, and consequently a wide variety of methods have been described. Many different columns and eluent mixtures have been used, which results in variation in separation efficiency. In addition, various labeling reagents have been used to increase hydrophobicity and detectability of the glycans. It was found that the hydrophobicity of the tag has a major influence on the separation and elution order of the different glycans. Moreover, this hydrophobicity also influences the contribution of the glycan itself to the retention.

Because of the relatively low hydrophobicity of PA, more structural information on the glycan can be derived. However, because of this low hydrophobicity, direct coupling with ESI-MS was less convenient. The more hydrophobic labels show a different elution order of glycans compared with PA-labeled glycans, but a uniform order could not be identified. Among other factors, the presence of an ion-pairing agent in the eluent, the type of column, the gradient, and the composition of the eluent have a substantial influence on the elution of glycans. Moreover, the presence of sialic acids or fucoses on the glycan also has a major influence on the retention. This influence is linkage specific, and in case of the fucose the influence also depends on whether the fucose it is located on the core or on the antenna of the *N*-glycan. In addition, the influence of sialic acids on the retention of glycopeptides is contradictory to the findings in released glycans. It appears that the exact influence of these monosaccharides is still incompletely understood, and additional research should be performed on this subject.

The quality of separation of the glycans is highly dependent on the complexity of the sample. In addition, a clustering of peaks is caused by the major contribution of the label to the retention, resulting in a low peak capacity. In complex samples, overlap between peaks of different glycan species has been observed. In fluorescence and UV-detection this causes difficulties with the identification of glycans, but for MS analysis this was often not a problem, as long as isomeric and isobaric species were separated from each other. The choice of the column, run time, and eluents influences the ability to separate isomers. In less complex samples, isomers were separated by reversed-phase chromatography. Unfortunately, no information on the elution order of these isomers was provided, which could have given insights into the retention of glycans on a reversed-phase stationary phase. The separation of isomers in complex samples could also be obtained by reversed-phase chromatography in a second dimension after HILIC separation. In addition, PGC chromatography, an emerging method for glycan separation, also has the capability to separate isomeric and isobaric glycans, also without derivatization. HILIC and PGC chromatography were compared with reversed-phase chromatography and from the data obtained it was concluded that both were better able to separate glycan species and their isomers. However, these methods have been proven to be less robust and reproducible than reversed-phase methods. Improvement of these properties might eliminate the need for reversed-phase glycan analyses in the future.

Further research should provide more information on the influence of the sialic acids and fucoses on the retention of the glycans and also of the glycopeptides. Especially the linkage and structure specificity of the fucoses should be investigated. In addition, the analysis of samples containing triantennary and tetraantennary glycans could give more insights into the influence of the sialic acid moieties.
